# Genomic diversity and antimicrobial resistance among non-typhoidal *Salmonella* associated with human disease in The Gambia

**DOI:** 10.1099/mgen.0.000785

**Published:** 2022-03-18

**Authors:** Saffiatou Darboe, Richard S. Bradbury, Jody Phelan, Abdoulie Kanteh, Abdul-Khalie Muhammad, Archibald Worwui, Shangxin Yang, Davis Nwakanma, Blanca Perez-Sepulveda, Samuel Kariuki, Brenda Kwambana-Adams, Martin Antonio

**Affiliations:** ^1^​ Medical Research Council Unit, The Gambia at London School of Hygiene and Tropical Medicine, The Gambia; ^2^​ Federation University, Melbourne, Australia; ^3^​ London School of Hygiene and Tropical Medicine, London, UK; ^4^​ University of California, Los Angeles, California, USA; ^5^​ University of Liverpool, Liverpool, UK; ^6^​ Kenyan Medical Research Institute, Nairobi, Kenya; ^7^​ University College London, London, UK

**Keywords:** antimicrobials, bacteraemia, gastroenteritis, multidrug resistance (MDR), whole genome sequencing (WGS), The Gambia, NTS

## Abstract

Non-typhoidal *Salmonella* associated with multidrug resistance cause invasive disease in sub-Saharan Africa. Specific lineages of serovars Typhimurium and Enteritidis have been implicated. Here we characterized the genomic diversity of 100 clinical non-typhoidal *Salmonella* collected from 93 patients in 2001 from the eastern, and in 2006–2018 from the western regions of The Gambia respectively. A total of 93 isolates (64 invasive, 23 gastroenteritis and six other sites) representing a single infection episode were phenotypically tested for antimicrobial susceptibility using the Kirby–Bauer disc diffusion technique. Whole genome sequencing of 100 isolates was performed using Illumina, and the reads were assembled and analysed using SPAdes. The *Salmonella in Silico* Typing Resource (SISTR) was used for serotyping. SNP differences among the 93 isolates were determined using Roary, and phylogenetic analysis was performed in the context of 495 African strains from the European Nucleotide Archive. *Salmonella* serovars Typhimurium (26/64; 30.6 %) and Enteritidis (13/64; 20.3 %) were associated with invasive disease, whilst other serovars were mainly responsible for gastroenteritis (17/23; 73.9 %). The presence of three major serovar Enteritidis clades was confirmed, including the invasive West African clade, which made up more than half (11/16; 68.8 %) of the genomes. Multidrug resistance was confined among the serovar Enteritidis West African clade. The presence of this epidemic virulent clade has potential for spread of resistance and thus important implications for systematic patient management. Surveillance and epidemiological investigations to inform control are warranted.

## Impact Statement

Invasive non-typhoidal *Salmonella* (NTS) is a leading cause of invasive bacterial disease in The Gambia including in patients with sickle cell disease. Previous studies have determined that NTS serovars Typhimurium and Enteritidis are prevalent with noted phenotypic serovar regional variations and multidrug resistance. The lack of accurate diagnostic microbiology facilities in the sub-Saharan region has resulted in a paucity of data and has hampered surveillance. Notwithstanding this, available data in the subregion have confirmed the presence of virulent multidrug-resistant lineages implicated in invasive disease. This study provides genomic insight into NTS serovars causing disease in The Gambia and confirms the presence of the virudent multidrug-resistant West African clade of *Salmonella* Enteritidis. This has important implications for patient management and control of antimicrobial resistance.

## Data Summary

Sequencing data are deposited in the NCBI sequence reads archive (SRA) under BioProject ID: PRJEB38968. The genome assemblies are available for download from the European Nucleotide Archive (ENA): https://www.ebi.ac.uk/ena/data/view. Accession numbers SAMEA6991082 to SAME6991180.

## **I**ntroduction

Non-typhoidal *Salmonella* (NTS) serovars are associated with foodborne gastroenteritis but can also cause severe disseminated infections dependent on the pathogen’s virulence and the host’s immune status [1, 2]. The pathogenic success of NTS serovars is directly linked to its plethora of virulence factors such as the cytolethal distending toxin gene (*cdt*B) and hence is responsible for differences in virulence traits [3, 4]. In addition, severity of disease is aided by host susceptibility, infectious dose and antimicrobial resistance. Globally, there are over 2800 serovars, some of which are adapted to non-human hosts [5]. The global annual estimate of *Salmonella* gastroenteritis is 93.8 million illnesses with an incidence of 1140 per 100 000 and 155 000 deaths [6]. Africa accounts for 2.4 million cases, an incidence of 320 per 100 000 and 4100 deaths [6]; this is a comparably lower burden when compared to other parts of the world. Despite the relatively lower prevalence of NTS gastroenteritis in Africa compared to the rest of world, the additional burden caused by invasive NTS (iNTS) disease in Africa makes it the continent with the highest NTS burden [7]. Most cases of *Salmonella* gastroenteritis in immunocompetent hosts are self-limiting and do not require antimicrobial therapy [8]. However, infections in infants, the elderly and immunocompromised patients do require antimicrobial treatments such as ciprofloxacin, third-generation cephalosporins or azithromycin [9].

iNTS are an important cause of extra-intestinal invasive disease and a major public health concern, with a high case fatality ratio of 20–25 % [1, 10–12] causing 49 600 annual deaths in sub-Saharan Africa (sSA) [13]. The predominant *Salmonella enterica* strains responsible for invasive disease in sSA are multidrug-resistant (MDR) lineages of *Salmonella enterica* serovar*s* Enteritidis (*S*. Enteritidis) and Typhimurium (*S*. Typhimurium) that are distinct from those circulating in other parts of the world [1, 14]. These lineages do not ellicit gut inflammation, thus facilitating an invasive phenotype similar to *S*. Typhi, and are adapted to the immunosuppressed human niche [1, 15]. Whole genome sequencing (WGS) has provided new insights into the host-adapted signatures associated with pathogenicity and metabolism of these Typhimurium lineages and Enteritidis clades, which are characterized by genomic degradation and different accessory genome [1, 11, 16].

Novel lineages of serovar Typhimurium causing invasive disease are confined mainly to immunocomprised patients in sSA and have mostly been identified as sequence type (ST) 313 [11]. The closely related serovar Typhimurium ST313 lineages I and II evolved independently around 52 and 35 years ago respectively, with clonal replacement by lineage II emerging with the acquisition of the chloramphenicol acetyltranferase (*cat*) resistance gene [11]. Both lineages have been shown to carry a *Salmonella* serovar Typhimurium virulence plasmid, commonly known as pSLT, which also encodes genes conferring resistance to common antimicrobials including tetracycline, sulfamethoxazole-trimethoprim and chloramphenicol [11, 16]. Recent data provide further evidence of evolution within ST313 and lineage replacement such as sub-lineage II.1 that harbours the IncHI2 plasmid and exhibits extensive drug resistance [17]. Furthermore, a new pan susceptible ST313 lineage (lineage III or ST313 L3) was identified as an intermediate between lineages I and II, which emerged in 2016 from Malawi, providing further evidence of *Salmonella* evolution [18].

Two related, but phylogenetically distinct epidemic clades of *Salmonella* serovar Enteritidis ST11, the West African clade and the Central/Eastern African clade, characterized by the presence of chloramphenicol acetyl resistance genes *catA1* and *catA2*, respectively, plus the incomplete set of *tra* genes, emerged between 1933 and 1945 [1]. The utility of second-line antimicrobials such as fluoroquinolones, azithromycin and extended-spectrum cephalosporins is limited in treating these emerging MDR strains [19].

In The Gambia, iNTS remains a leading cause of invasive disease in both eastern and western regions [20–22]. Regional serovar variation and emerging MDR has been previously described in The Gambia, with *Salmonella* serovar Typhimurium predominating in the western region and *Salmonella* serovar Enteritidis confined to the eastern region [23, 24]. In this context, we performed WGS analysis of clinical NTS isolates from two different regions to determine genotypes and antimicrobial resistance genes for understanding the genomic epidemiology of serovars associated with disease from the two regions of The Gambia in the context of virulent lineages circulating around the sub-region. Regional epidemiological surveillance is critical to monitor circulating serovars as understanding geographical serovar diversity is crucial in the management of patients. The resulting analysis can be used to help guide clinical management and control of NTS diseases in The Gambia.

## Methods

### Study setting and population

**Fig. 1.**
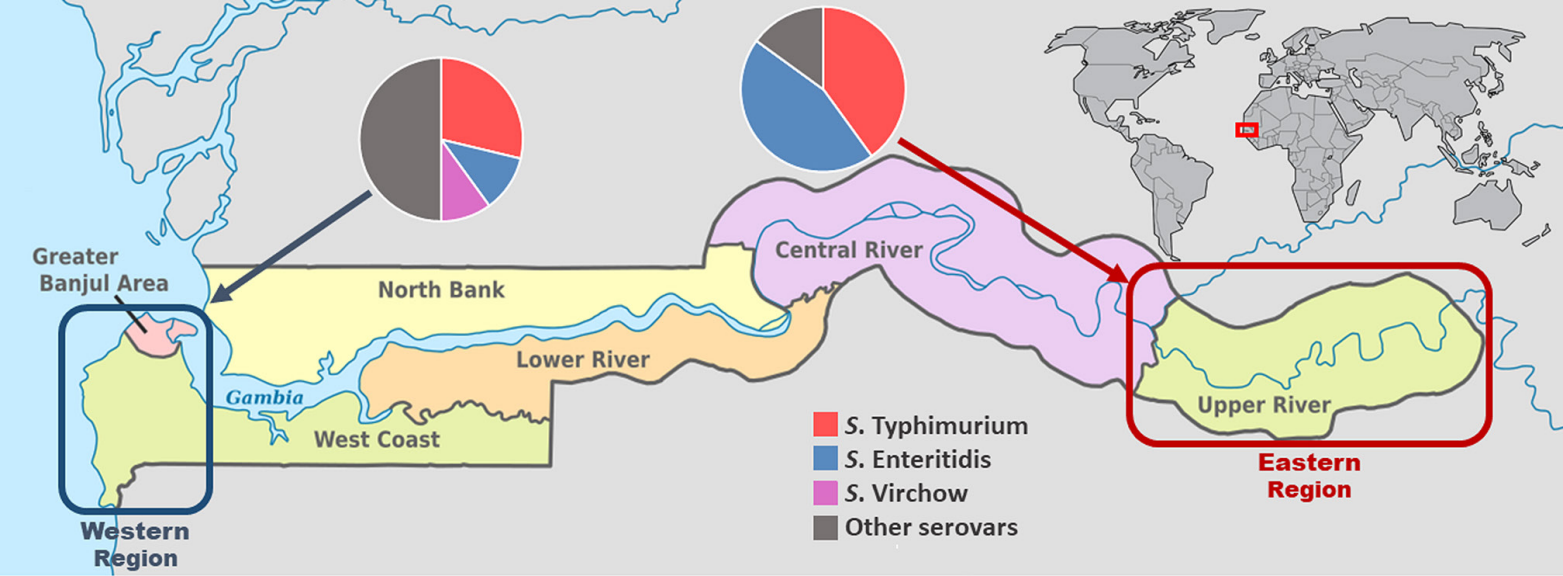
Map of The Gambia showing the geographical locations of the two regions sampled in this study (western region, dark blue square; eastern region, burgundy square) and pie charts demonstrating the percentage prevalence of major NTS serovars recovered (large pie chart) in each region.

The study was conducted at the Medical Research Council Unit The Gambia (MRCG) at the London School of Hygiene and Tropical Medicine (LSHTM) using clinical NTS isolates from patients in the eastern (Upper River Region, a relatively rural setting) and western (West Coast Region and Greater Banjul Area, an urban setting) regions of The Gambia (Fig. 1). The eastern region, located on the far east side of the river Gambia, is the commercial centre and a busy economic hub, with an estimated population of 200 000 people. It is an important transit point for merchandise and people going into eastern Senegal, Mali and Guinea Conakry. The western region is densely populated, with a population of over 1 million people including the capital city, Banjul (Fig. 1) [25]. Malaria has declined in recent years but remains endemic with peak transmission occurring from July to November [26]. Malnutrition remains a problem, with the prevalence of underweight, stunting and wasting among children under 5 years old estimated at 16.4, 25.0 and 4.3%, respectively [27]; HIV (human immunodeficiency virus) prevalence among adults aged 15–49 years is estimated at 2.1 % [28].

The MRCG hospitals in both regions provide primary and secondary-level care to sick individuals from the surrounding population with complicated cases referred to the main tertiary government hospitals. MRCG sites Basse and Fajara are the only health facilities in The Gambia where diagnostic microbiological cultures are routinely carried out on patients with suspected bacterial infections. In Fajara (western region), blood and cerebrospinal fluid (CSF) samples are routinely collected for bacterial culture from patients presenting with suspected sepsis and treated with ampicillin and gentamicin, while those with suspected meningitis are treated with ceftriaxone prior to laboratory confirmation. Stool samples are sent for bacterial culture for those with suspected gastroenteritis. In the Basse site (eastern region), patient samples were from enrolled particpants in a clinical trial who became ill during the course of the study and whose clinical samples were sent for routine testing, using similar methods as above.

### Sample collection, microbiological procedures and antimicrobial susceptibility testing

The study evaluated 100 clinical NTS isolates from 93 patients with NTS bacteraemia or meningitis (invasive, 68/100), diarrhoea disease (gastroenteritis, 26/100) or other focal infections (6/100) visiting two hospitals in the eastern and western regions of The Gambia (Table 1). Seven patients had multiple samples collected during the same infection episode, of which three were concurrent bacteraemia and gastroenteritis, two had bacteraemia with meningitis whilst two had bacteraemia with two sampling episodes (Table S2). Duplicate samples during the same infection episode were excluded from further analysis, and the isolate from the most invasive site was maintained. For example, for patients with isolates obtained from blood and stool samples, the blood isolate was kept.

**Table 1.** Baseline characteristics of Gambian NTS disease patients from whom isolates were cultured for use in this study

		*n* (%)	Eastern region	Western region
Patients		93	18	75
Age range	0–4 years	42 (45.2)	4 (25.0)	38 (50.7)
	5–14 years	21 (22.6)	12 (65.0)	9 (12.0)
	≥15 years	26 (27.9)	2 (10.0)	24 (32.0)
	Unknown	4 (4.3)	0	4 (5.3)
Gender	Male	51 (54.3)	10 (55.6)	41 (54.7)
	Female	38 (41.5)	7 (38.9)	31 (41.3)
	Unknown	4 (4.2)	1 (5.5)	3 (4.0)
**Bacterial isolates**		**100**	**20**	**80**
Source	Invasive disease	68 (68.0)	19 (95.0)	49 (61.2)
	Gastroenteritis	26 (26.0)	1 (5.0)	25 (31.3)
	Other	6 (6)	0	6 (7.5)
Serovars	*S*. Enteritidis	18 (18.0)	9 (45.0)	9 (11.2)
	*S*. Typhimurium	31 (31.0)	8 (40.0)	23 (28.8)
	*S*. Virchow	8 (8.0)	0	8 (10.0)
	Other serovars*	43 (43.0)	3 (15.0)	40 (50.0)

*Shown in Table S1.

All isolates were stored in 15 % (v/v) glycerol at −70 °C. The isolates were grown on MacConkey agar overnight at 37 °C in the Clinical Microbiology Laboratory. The laboratory is accredited to Good Clinical Laboratory Practice (GCLP; 2010) and ISO15189 (2015) as previously described [23]. Antimicrobials were tested according to the 2017 Clinical Laboratory Standard Institute (CLSI) guidelines [29]. Antimicrobial susceptibility of the 93 isolates to amoxicillin-clavulanate, ampicillin, cefotaxime, cefoxitin, ceftazidime, ceftriaxone, cefuroxime, chloramphenicol, ciprofloxacin, gentamicin, nalidixic acid, sulfamethoxazole-trimethoprim and tetracycline was tested on Mueller–Hinton agar (MHA) using the Kirby–Bauer disc diffusion method. Streptomycin, fosfomycin and azithromycin were not phenotypically tested for the following reasons; streptomycin lacks clinical breakpoints, fosfomycin is indicated for urinary infections and azithromycin breakpoints are based on minimum inhibitory concentration (MIC) data. Antimicrobial agents were from BD Oxoid and *Escherichia coli* (ATCC 25922) with known antimicrobial resistance (AMR) profile was used as a quality control.

### DNA extraction and whole genome sequencing

Genomic DNA was extracted and sequenced in two locations. The majority of isolates (*n*=67) were processed at the University of Liverpool (UK) and sequenced at the Earlham Institute (UK). DNA extraction and sequencing were carried out using an optimized method for large-scale sequencing [30], including the bespoke LITE (Low Input, Transposase Enabled) pipeline for library construction, and Illumina HiSeq sequencing technology. The remaining isolates (*n*=33) were processed and sequenced at the MRCG. DNA extraction was performed using the QIAamp DNA Mini kit (Qiagen) to extract the DNA from 1.5 ml of an overnight culture grown in triple soya broth (TSB) from BD Oxoid at 37 °C, according to the manufacturer’s instructions, and quantified using a Qubit fluorometer (ThermoFisher; Qubit dsDNA HS Assay). Libraries were prepared using the Nextera XT kit using the Illumina MiSeq system. All isolates (*n*=100) were sequenced using the 2×150 bp read protocol.

### Genome assembly and *in silico* analysis

The quality of raw Illumina reads was assessed using FASTQC (v0.11.5) [31]. An average quality Phred score (Qscore) above 30 was used as a cut-off. Paired-end reads were trimmed using Trimommatic (v0.39) [32] and assembled into contigs using SPAdes (v1.0.4), with default settings [33]. *In silico* serotyping was predicted using the *Salmonella in Silico* Typing resource (SISTR) [34]. eBurst Groups (eBGs) were assigned using the Enterobase platform (http://enterobase.warwick.ac.uk) [35], which is based on the allelic identity that accounts for homologous recombination, defined as closely related natural genetic clusters/populations of two or more STs connected by pairwise identity or single-locus variants [36].

### Phylogenetic analysis

Assembled contigs were annotated using Prokka v1.14.6 [37]. The core genome was determined using Roary v3.13.0 [38], taking the GFF files from Prokka as input with default settings. A core genome alignment was created using roary, which uses mafft to align the individual core genes (v7.467, -e --mafft) [39] . The whole core-gene alignment was used to create a maximum-likelihood phylogeny using IQ-TREE with the general time-reversible (GTR) model with gamma distributed rate variation among sites (G) (v1.6.12, -m GTR +G) (http://www.iqtree.org/release/v1.6.12). Serovar-specific phylogenies were generated by running IQ-TREE with the same parameters on the same core-genome alignment subsetted to contain only *S*. Enteritidis and *S*. Typhimurium isolates. The phylogenetic tree was visualized and annotated using the interactive Tree Of Life (iTOL) [40]. iTOL annotation input files for the tree were generated using custom python scripts (https://github.com/jodyphelan/itol-config-generators). AMR, plasmids and virulence genes were detected by using abricate (https://github.com/tseemann/abricate) with databases from ResFinder [41], PlasmidFinder [42] and virulence factor gene database (VFDB) [43] with a minimum coverage and nucleotide identity of 98 % as the cut-off. Publicly available data were downloaded from the European Nucleotide Archive (ENA) to use as context for other African strains. All sequence reads from the African samples belonging to the NCBI:taxid149539 (the unique NCBI number that applies to *S*. Enteritidis) were downloaded and assembled/annotated using the same methods as detailed above. The core-genome phylogeny was reconstructed using the same methods as outlined above. Sequence data have been deposited in the NCBI sequence reads archive (SRA) under BioProject PRJEB38968 (isolate accessions are available in Table S3)

### Statistical analysis

We compared descriptive relationships between groups based on serovar data using logistic regression with measures of association expressed as odds ratios. We summarized our binary variables with frequencies and percentages whilst continuous variables were summarized using medians and interquartile range (IQR) as appropriate. No power calculations were performed and an alpha value of 0.1 was considered statistically significant. All statistical analyses were performed in Stata, v13.1 (StataCorp 2013; Stata Statistical Software: Release 13).

## Results

### Isolate source, associated disease syndrome and regional serovar differences

One hundred isolates were recovered from clinical samples in 2001 from 18 patients in the eastern region (*n*=20) and from 75 patients between 2006 and 2018 in the western region (*n*=80) of The Gambia (Table 1). Isolates from the eastern region were predominantly from invasive disease (17 blood and two CSF) with only one gastroenteritis (stool) source. Isolates from the western region were from invasive disease (48 blood and one CSF), gastroenteritis (25 stool) and other focal non-invasive infections (five abscesses/pus and one urine). In total, 93/100 isolates considered as single-episode infection from patients with a median age range of 5–14 years were further analysed. *Salmonella* serovars other than *S*. Enteritidis and *S*. Typhimurium were primarily responsible for gastroenteritis (17/23; 73.9 %), whilst *S*. Typhimurium (26/64; 30.6 %) and *S*. Enteritidis (13/64; 20.3 %) were the leading serovars associated with invasive disease, being 15 times and almost three times as likely to be recovered from an invasive site than gastrointestinal site, respectively (Table S4).

### Sequence types and eBurst groups

All 31 *S*. Typhimurium were eBG1, of which 29 were sequence type ST19, and two were with one or two allelic variants. All 16 *S*. Enteritidis belonged to eBG4, of which 15 were ST11 and one was ST1925. *Salmonella* serovar Virchow was in eBG9 and assigned to ST181, ST755 and ST841. The four *Salmonella* serovar Hull isolates belonged to eBG330 and were assigned ST1996. *Salmonella* serovar Stanleyville eBG79 (ST339), *Salmonella* serovar Poona eBG46 (ST308) and *Salmonella* serovar Give eBG67 (ST516) all belonged within a single sequence type.

### AMR genes, AMR phenotypes, plasmid replicons and virulence genes

**Fig. 2.**
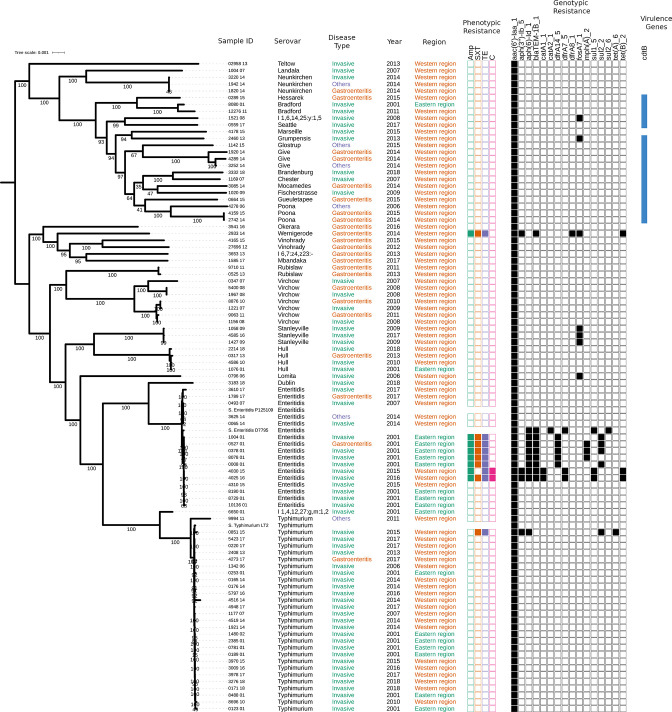
Phylogenetic tree reconstructed with IQ-TREE using the core genome of strains sequenced for this study showing: sample IDs, *Salmonella* serovar, disease type, year of isolation and region. The tree was built using maximum-likelihood methods implemented in IQ-TREE followed by mid-point rooting. The presence of phenotypic (solid coloured squares), genotypic resistance genes (solid black squares) and the virulence gene *cdtB* is also shown. Bar, 0.001 changes per site. Numbers on the branches represent the ultra fasta bootstrap approximation.

AMR genes belonging to eight classes of antimicrobials, plus an aminoglycoside cryptic gene, *aac(6')-Iaa_1*, were present in all strains (Fig. 2). Other AMR genes were harboured by 16/93 (17.2 %) isolates and conferred resistance to aminoglycosides (*aph_*3_Ib and *aph_*6_Id; *n*=12), tetracyclines (*tet_*A and *tet_*B; *n*=9), trimethoprim (*dfr*A14*, dfr*A7 and *dfr*A8; *n*=8), sulfamethoxazole (*sul2* and *sul1*; *n*=7), ampicillin (*blaTEM-*1B; *n*=8), fosfomycin (*fos*A7_1; *n*=7), azithromycin (*mph_*A; *n*=3) and chloramphenicol (*cat*A1_1; *n*=2) (Table 2). The presence of the two aminoglycoside resistance genes (*aph_3_Ib* and *aph_6_Id*) only conferred resistance to streptomycin. The presence of three or more AMR genes was found in 9/93 (9.7 %) isolates, 7/9 (77.8 %) of which belonged to the serovar *S*. Enteritidis.

**Table 2.** Summary of genotypic and phenotypic characteristics of serovars with resistance genes

Strain	ST	Sample	Region	Resistance gene(s)	Phenotypic resistance
0796_06; *S*. Lomita	3039	Blood	Western region	fosA7_1	Not tested
1058_09; *S*. Stanleyville	339	Blood	Western region	fosA7_1	Not tested
1427_09; *S*. Stanleyville	339	Blood	Western region	fosA7_1	Not tested
4585_16; *S*. Stanleyville	339	Blood	Western region	fosA7_1	Not tested
1503_08; 1,6,14,25:y:1,5	6046	Blood	Western region	fosA7_1	Not tested
2460_13; *S*. Grumpensis	2060	Blood	Western region	fosA7_1	Not tested
2933_14: *S*. Wernigerode	2271	Stool	Western region	fosA7_1, blaTEM-1B_1, aph_3_Ib_5, dfrA8_1, tet_B__2	Ampicillin, sulfamethoxazole-trimethoprim, tetracycline
3625_14; *S*. Enteritidis	1925	Urine	Western region	tet_A__6	Tetracycline
0851_15: *S*. Typhimurium	19	Blood	Western region	tet_A__6, sul2_2, aph_6_Id_1, aph_3_Ib_5	Tetracycline, sulfamethoxazole-trimethoprim
0008_01: *S*. Enteritidis	11	Blood	Eastern region	aph_6_Id_1, blaTEM-1B_1, dfrA14_5, sul2_2	Ampicillin, sulfamethoxazole-trimethoprim
1004_01; *S*. Enteritidis	11	Blood	Eastern region	blaTEM-1B_1, dfrA14_5, tet_A__6, sul2_2, aph_6_Id_1	Ampicillin, sulfamethoxazole-trimethoprim, tetracycline
8078_01; *S*. Enteritidis	11	Blood	Eastern region	blaTEM-1B_1, dfrA14_5, tet_A__6, aph_6_Id_1, mph_A__2	Ampicillin, sulfamethoxazole-trimethoprim, tetracycline
0378_01; *S*. Enteritidis	11	Blood	Eastern region	blaTEM-1B_1, dfrA14_5, tet_A__6, sul2_2, aph_6_Id_1, mph_A__2	Ampicillin, sulfamethoxazole-trimethoprim, tetracycline
0527_01; *S*. Enteritidis	11	Stool	Eastern region	blaTEM-1B_1, dfrA14_5, tet_A__6, sul2_2, aph_6_Id_1, mph_A__2	Ampicillin, sulfamethoxazole-trimethoprim, tetracycline
4025_16; *S*. Enteritidis	11	Blood	Western region	blaTEM-1B_1, dfrA7_5, catA1_1, sul1_5, tet_B__2, aph_3_Ib_5, aph_6_Id_1	Ampicillin, sulfamethoxazole-trimethoprim, tetracycline, chloramphenicol
4030_15; *S*. Enteritidis	11	Blood	Western region	blaTEM-1B_1, dfrA7_5, catA1_1, sul1_5, tet_B__2, aph_3_Ib_5, aph_6_Id_1	Ampicillin, sulfamethoxazole-trimethoprim, tetracycline, chloramphenicol

Phenotypic resistance to multiple antimicrobial classes was also observed for 9/93 (9.7 %) isolates, correlating with the presence of resistance genes to tetracycline, ampicillin, sulfamethoxazole-trimethoprim and chloramphenicol (Table 2). Streptomycin, fosfomycin and azthromycin were not phenotypically tested due to a lack of clinical relevance or appropriate testing method (Fig. 2). Resistance to ampicillin, sulfamethoxazole-trimethoprim and tetracycline was 59, 22 and 29 times more likely for *S*. Enteritidis than all other serovars combined (Table S5).

**Fig. 3.**
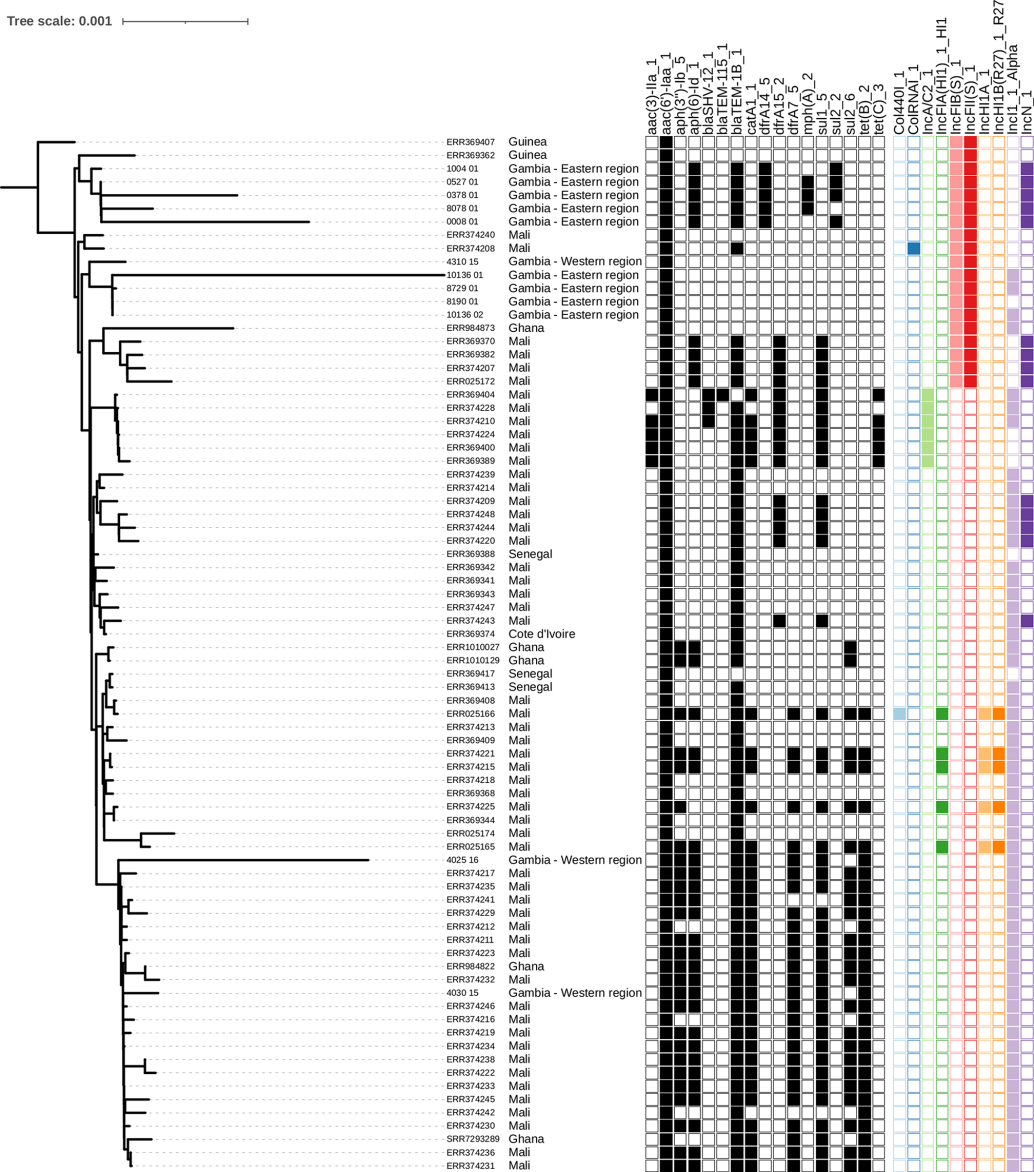
Phylogenetic tree showing presence and absence of AMR-associated genes of *S*. Enteritidis within the West African clade. The tree was built by applying maximum-likelihood phylogenetic reconstruction implemented by IQ-TREE followed by mid-point rooting. Each branch is labelled with either isolate name (this study) or accession number (reference strains), followed by country of isolation. Presence (filled squares) or absence (empty squares) of AMR genes and plasmid replicons are displayed for each isolate. Bar, 0.001 maximum-likelihood genetic distance estimated by IQ-TREE.

Nineteen different plasmid replicons were detected in 61/93 (65.6 %) isolates; seven isolates harboured one plasmid replicon, 35 harboured two, 15 harboured three and four harboured four plasmid replicons (Table S6). The most common plasmid types were IncFII (*n*=50) and IncFIB (*n*=45), harboured by all *S*. Typhimurium and all but the two chloramphenicol-resistant *S*. Enteritidis. The IncN_1 plasmid was associated with MDR, including azithromycin resistance, and was only found in *S*. Enteritidis from the eastern region (Fig. 3). The IncN plasmid is reported to be associated with beta-lactam, streptomycin and sulphonamide resistance [44]. Interestingly, the IncI1_Alpha was harboured by the two chloramphenicol MDR *S*. Enteritidis strains from the western region and the susceptible strains from the eastern region. No plasmid replicons were detected in serovars Bradford, Hull, Stanleyville, Rubislaw, Vinohrady or 1,4,12,27:g,m:1,2. In addition, the virulence gene *cdtB*, which is known to increase virulence and was previously thought to be restricted in *S*. Typhi, was present in 18 isolates.

### Phylogenetic analysis

**Fig. 4.**
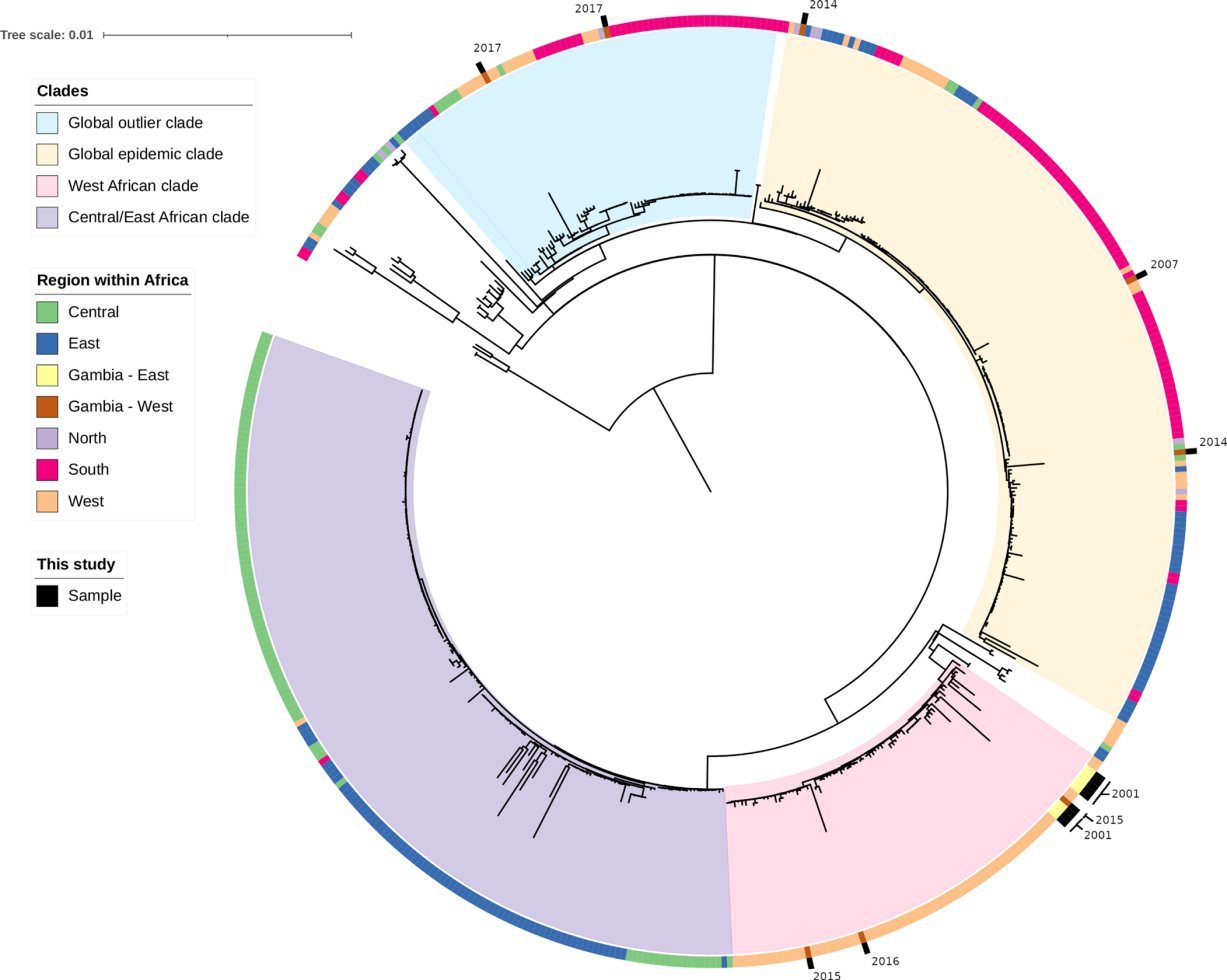
The phylogeny of *S*. Enteritidis from the African region. The maximum-likelihood tree was reconstructed using IQ-TREE; bar, 0.01 genetic distance. The tree was rooted using the mid-point rooting technique. The majority of the isolates from this study are placed within the West African clade. Samples from this study are labelled on the outer ring.

SNP analysis showed the isolates are clustered into respective serovars (Fig. 2). *S*. Typhimurium ST19 comprised one cluster whilst *S*. Enteritidis fell within three clades (Figs S1 and S2). To put our data within the wider regional context, we compared 16 non-duplicate *Salmonella* serovar Enteritidis strains from our study to 495 available African *S*. Enteritidis genomes (Fig. 4). Our analysis revealed the *Salmonella* serovar Enteritidis from this study clustered into three clades (Fig. 4): 11/16 (eastern region *n*=8 and western region *n*=3) clustered within the West African clade, known to cause invasive diseases and to carry the *cat*A1 gene [1]. Two clustered within the global outlier clade and were isolated from blood (*n*=1) and stool (*n*=1), and three clustered within the global epidemic clade isolated from blood (*n*=2) and urine (*n*=1). Seven of 11 (66.6 %) isolates within the West African clade were MDR (five from the eastern region and two from the western region). Only the two MDR isolates from the western region carried the *catA1* gene (Fig. 3) and all but one of the isolates in the West African clade were isolated from blood. Among all the analysed strains within the West African clade, the azithromycin resistance *mph*_A_2 gene was only harboured by strains from the eastern region (Fig. 3).

## Discussion

We used phylogenetic analysis to confirm the circulation of a diverse range of NTS serovars including the epidemic *Salmonella* serovar Enteritidis West African clade in The Gambia, as far back as 2001. This clade is associated with high mortality, isolates harbour MDR genes and exhibit genome degradation, thus adapting to an invasive lifestyle [1, 45]. Host factors such as immune suppression, age and malaria infection are possibly contributing to these invasive virulent lineages [1, 11]; however, these host factors were not assessed in this study. Notwithstanding this, the majority of patients who had isolates from the West African clade were mainly from the eastern region, which has a higher rate of HIV infection [46] and malaria prevalence [47]. Although the eastern region is less populated than the western region, it is characterized by an important point for movement of merchandise and people into neighbouring West African countries where HIV and malaria are also of great concern. In addition, recent molecular analysis of NTS by other authors has confirmed the presence of ST313 in this region [48]. This warrants further epidemiological investigations and surveillance as it has important implications for treatment. Interestingly, one-third of the West African clade *S*. Enteritidis in this study did not harbour resistance genes and were phenotypically susceptible, as opposed to the high MDR in this clade reported by other studies [1, 45]. This could be an interesting adaptation mechanism that requires investigation for the evolution for *S*. Enteritidis, as described for susceptible *S*. Typhimurium lineages [18].

Remarkably, this study only found *S*. Typhimurium ST19 causing invasive NTS disease as opposed to the predominant ST313 virulent lineage, endemic in other parts of sSA [11, 18, 49]. Although ST19 still remains associated with invasive disease in the subregion, it is usually more susceptible to antimicrobials [18]. The absence of ST313 may be due to the overall low prevalence of HIV in The Gambia compared to other regions in sSA, and difference in serovar distribution [23, 28, 46]. This highlights the unique epidemiological differences seen in the Gambia regarding NTS. Furthermore, a study in Brazil reported that the *S*. Typhimurium ST19 lineage has evolved similar to ST313 restricted to invasive disease [50]. This unique pathogenesis therefore warrants further comparative genomic and epidemiological investigations into the *S*. Typhimurium ST19 lineage [20].

Two-thirds of serovars responsible for gastroenteritis were serovars other than *S*. Typhimurium and *S*. Enteritidis. The difference in the number of isolates of serovars causing gastroenteritis as opposed to iNTS may be due to sampling bias, with patients presenting to hospital with obvious clinical syndromes necessitating sampling. Notwithstanding this, other serovars were still an important cause of invasive disease and responsible for up to 30 % of infections. To further put this into context, a recent population-based study found serovars other than *S*. Enteridis and *S*. Typhimurium as important causes of iNTS in the eastern region. The study confirmed *S*. Dublin as the most common serovar [48] as opposed to *S*. Virchow found in our study for the western region. In addition, the majority of serovars in this study belong to the *S. enterica* subspecies *enterica* clade B, which are specialized and adapted to the gastrointestinal niche, and hence likely to cause such infections [51]. Similarly, a population-based study in The Gambia over a decade on children with gastroenteritis living in close proximity to animals found similar clade B serovars among the NTS isolated and further discounted zoonotic transmission [52], in contrast to what was observed in Kenya and Malawi with zoonotic transmission from pigs [53].

Furthermore, the GEMs (Global Enteric Multicentre Study) population-based case-control study of acute moderate to severe diarrhoea among children under 5 years determined that NTS is prevalent, although not a major cause of gastroenteritis in Africa [54, 55]. Molecular analysis of the NTS confirmed the presence of ST313 asymptomatic carriage and in diarrhoeal patients, further highlighting that sequence type is not just associated with invasive disease and demonstrating anthroponotic transmission [55]. A huge gap exists regarding transmission dynamics of NTS in sSA [56], and thus more insight is needed in understanding the relationship between NTS gastroenteritis and iNTS disease. However, it may also suggest that NTS gastroenteritis may not be a predisposition to iNTS in The Gambia, and other transmission pathways need to be investigated. Notwithstanding, iNTS remains a leading cause of bacteraemia in The Gambia [20, 21] albeit with a general decline associated with the decline in incidence of malaria [57].

This study has shown epidemiological differences in iNTS serovars associated with disease in The Gambia. Previous studies (including a recent study) have highlighted a geographical association of *Salmonella* serovars between the two regions [23, 24, 48], indicating a possible region-specific epidemiological pattern of NTS in The Gambia. However, the time difference in sampling between the two regions may confound the variance in location and warrant further investigation. The changing disease pattern of NTS in sSA, associated with specific lineages of *S*. Typhimurium and *S*. Enteritidis, remains a major concern and warrants surveillance [1, 14, 58]. Importantly, three cases of *Salmonella* bacterial meningitis were included in this study, all of which were found in patients under 10 years old. Although rare, NTS meningitis has been reported elsewhere in Africa, and is often associated with high case fatality [59, 60]. Therefore, NTS needs to be considered in the differential diagnosis of bacterial meningitis following post-vaccine declines in the prevalence of *Haemophilus influenzae* type B, *Neisseria meningitidis* and pneumococcal meningitis [61, 62].

MDR was confined within *Salmonella* serovar Enteritidis mainly isolated from the eastern region and noted for first-line antibiotics such as ampicillin, sulfamethoxazole-trimethoprim, tetracycline and chloramphenicol. A recent study from this region also found higher resistance in *S*. Enteritidis than other serovars, confirming our findings [48]. Nonetheless, no fluroquinolone or cephalosporin resistance was identified, implying these drugs might still be effective in The Gambia. Notwithstanding this, the emergence of the azithromycin resistance gene *mph_*A requires further monitoring as a recommended drug of choice for iNTS [63]. The cryptic aminoglycoside resistance gene *aac(6')-Iaa* was present in all serovars, including pan-susceptible isolates, but is known to have no evolutionary potential to increase antimicrobial resistance [64], as with other AMR determinats that warrant surveillance. This highlights the potential of using genome-based AMR prediction to monitor AMR determinants for emerging resistance. Our study did not phenotypically test streptomycin susceptibility, which lacks clinical breakpoints and is not used in the treatment of infections. In addition, the streptomycin resistance genes were frequently found to lack expression [65]. While the development of AMR has been mainly attributed to antibiotic misuse in humans and animals, evidence has shown that environmental factors such as poor sanitation, hygiene and access to clean water may be equally responsible for driving resistance in low- and middle-income countries [66, 67].

Geographical differences seen in AMR may suggest differences in selective pressure and ecological factors, thus highlighting the need for location-specific control measures. Factors such as the use of antimicrobials in food-producing animals are contributing to the emergence and dispersal of AMR in humans [68]. Our findings are consistent with other studies that show NTS serovar differences in geographical locations within the same country [68, 69]. A correlation between phenotypic and genotypic resistance was also observed, and the *IncN*-type plasmid was strongly associated with resistance and was found only in MDR *S*. Enteritidis, thus requiring closer surveillance. This plasmid is associated with dissemination of antimicrobial resistance with high potential of spread [44]. Although the limitation of using short read sequences to predict large plasmids remain, the short read predictions by PlasmidFinder use previously assembled contigs in databases to improve accuracy [42].

We found virulence genes which were not identified for specific phenotypic traits as this was beyond the scope of this study. However, the presence of the *cdtB* virulence gene previously thought to be restricted to *S*. Typhi in some serovars [3, 51] is an important virulence factor that warrants further investigation. The pathogenic success of NTS serovars is directly linked to their virulence factors aided by host susceptibility, serovar fitness, infectious dose and AMR [70]. Therefore, further characterization of the virulence genes are needed to understand the clinical implications. Moreover, the *cdtB* virulence gene has been found to be an important virulence marker in a recent study from a rural region of The Gambia [48].

There are several limitations to this study. First, the isolates were collected at different time points, with a lag of up to 18 years between the two different regions, which may lead to missing temporal differences. Notwithstanding this, the diversity of AMR between serovars and geographical regions highlights the need for real-time surveillance to detect regional differences and potential spread of resistance as well as region-appropriate interventions to effectively combat AMR. Second, relatively few isolates were analysed from only two regions due to limited microbiology capacity, and therefore our results may not reflect the entirety of strains and lineages of NTS in The Gambia.

In conclusion, this study provides evidence for the presence of the MDR *S*. Enteritidis epidemic West African clade in The Gambia. These findings have important implications for antimicrobial prescription policies and regional surveillance of NTS disease for better disease management and prevention.

## Supplementary Data

Supplementary material 1
