# Supplementary material for: Genomic diversity and antimicrobial resistance among non-typhoidal Salmonella associated with human disease in The Gambia
Source: Microb Genom. 2022 Mar 18;8(3):000785. doi: 10.1099/mgen.0.000785 (PMC9176284; doi:10.1099/mgen.0.000785)

Supplementary Table 1: Diversity of other serovars isolated among Gambian NTS in the study, frequency, and associated disease syndrome.

Other Serovar	*Number*	*Disease syndrome*
S. Bradford	2	Bacteraemia
S. Bradenburg	1	Bacteraemia
S. Chester	1	Bacteraemia
S. Dublin	1	Bacteraemia
S. Fischerstrasse	1	Bacteraemia
S. Give	3	Gastroenteritis, Abscess
S. Glostrup	1	Abscess
S. Grumpensis	1	Bacteraemia
S. Gueuletapee	1	Gastroenteritis
S. Hessarek	1	Gastroenteritis
S. Hull	4	Bacteraemia, Gastroenteritis
S. Landala	1	Bacteraemia
S. Lomita	1	Bacteraemia
S. Marseille	1	Bacteraemia
S. Mbandaka	1	Gastroenteritis
S. Mocamedes	1	Gastroenteritis
S. Neunkirchen	3	Abscess, Bacteraemia, Gastroenteritis
S. Okerara	1	Gastroenteritis
S. Poona	3	Abscess, Gastroenteritis
S. Rubislaw	2	Gastroenteritis
S. Seatlle	1	Bacteraemia
S. Stanleyville	3	Bacteraemia
S. Teltow	1	Bacteraemia
S. Vinohrady	2	Gastroenteritis
S. Wernigerode	1	Gastroenteritis
I 1,4,12,27:g,m:1,2	1	Bacteraemia
I 1,6,14,25:y:1,5	2	Bacteraemia
I 6,7:z4,z23:-	1	Gastroenteritis

Supplementary table 2: Patients with duplicate sampling NTS

Patient	Sex	Age range (yrs)	Infection source	Serovar	Region	Date isolated
**1**	Male	0-4	Bacteraemia	*S.* Enteritidis	western	19/9/2017
			Gastroenteritis	*S.* Enteritidis		14/9/2017
2	Female	35-39	Bacteraemia	*S.* Typhimurium	western	1/1/2014
			Gastroenteritis	*S.* Typhimurium		1/1/2014
3	Male	0-4	Bacteraemia	*S.* Virchow	western	1/3/2009
			Meningitis	*S.* Virchow		1/3/2009
4	Male	30-34	Bacteraemia	*S.* Typhimurium	western	1/11/2006
			Gastroenteritis	*S.* Typhimurium		1/11/2006
5	Female	0-4	Bacteraemia	1,6,14,25:y:1,5	western	1/10/2008
			Bacteraemia	1,6,14,25:y:1,5		1/10/2008
6	Male	5-9	Bacteraemia	*S.* Enteritidis	eastern	1/10/2001
			Bacteraemia	*S.* Enteritidis		1/10/2001
7	Male	0-4	Bacteraemia	*S.* Typhimurium	eastern	1/12/2001
			Meningitis	*S.* Typhimurium		1/12/2001

Supplementary Table 3. Strain metadata and accession numbers

Sample ID	Region	Serovar	Serogroup	ST	Day	Month	Year	Source	Disease	Age Range	Adult Child	Gender	Assembly accession numbers (PRJEB38968)	Reads accession numbers (PRJEB35182)	Reads accession numbers (PRJEB38968)	Associated barcode (PMID: 34930397)
12276_11	Western	Bradford	B	4253	10	10	2011	Blood	Invasive	0-4	Child	Male	GCA_903930945	SAMEA6142017		FD01843878
8080_01	Eastern	Bradford	B	4253	11	12	2001	Blood	Invasive	10-14	Child	Male	GCA_903930955	SAMEA6142009		FD01843868
3332_18	Western	Brandenburg	B	65	8	8	2018	Blood	Invasive	0-4	Child	Female	GCA_903931115		ERR7987340	
1169_07	Western	Chester	B	411	11	9	2007	Blood	Invasive	0-4	Child	Female	GCA_903931045	SAMEA6142727		FD01845442
3183_18	Western	Dublin	D1	10	30	7	2018	Blood	Invasive	0-4	Child	Female	GCA_903931385		ERR7987342	
3610_17	Western	Enteritidis	D1	11	19	9	2017	Blood	Invasive	0-4	Child	Male	GCA_903931395		ERR7987344	
0378_01	Eastern	Enteritidis	D1	11	27	2	2001	Blood	Invasive	10-14	Child	Male	GCA_903931545	SAMEA6143126		FD01846159
0527_01	Eastern	Enteritidis	D1	11	19	3	2001	Stool	Gastroenteritis	5-9	Child	Male	GCA_903931475	SAMEA6143140		FD01846175
1004_01	Eastern	Enteritidis	D1	11	15	3	2001	Blood	Invasive	0-4	Child	Female	GCA_903931525	SAMEA6143119		FD01846144
10136_01	Eastern	Enteritidis	D1	11	1	10	2001	Blood	Invasive	5-9	Child	Male	GCA_903931575	SAMEA6142004		FD01843860
1789_17	Western	Enteritidis	D1	11	6	6	2017	Stool	Gastroenteritis		Adult	Male	GCA_903931405		ERR7987345	
0493_07	Western	Enteritidis	D1	11	24	4	2007	Blood	Invasive	0-4	Child	Male	GCA_903931445	SAMEA6142675		FD01845379
0065_14	Western	Enteritidis	D1	11	7	1	2014	Blood	Invasive	20-24	Adult	Male	GCA_903931425	SAMEA6142008		FD01843865
3625_14	Western	Enteritidis	D1	1925	7	10	2014	Urine	Others	30-34	Adult	Male	GCA_903931435	SAMEA6142032		FD01843895
4030_15	Western	Enteritidis	D1	11	19	10	2015	Blood	Invasive	30-34	Adult	Female	GCA_903931465	SAMEA6142013		FD01843874
4310_15	Western	Enteritidis	D1	11	10	11	2015	Blood	Invasive	10-14	Child	Male	GCA_903931535	SAMEA6142040		FD01843906
8078_01	Eastern	Enteritidis	D1	11	1	4	2001	Blood	Invasive	5-9	Child	Male	GCA_903931515	SAMEA6143114		FD01846129
8190_01	Eastern	Enteritidis	D1	11	8	12	2001	Blood	Invasive	15-19	Child	Female	GCA_903931565	SAMEA6143152		FD01846192
8729_01	Eastern	Enteritidis	D1	11	1	1	2001	Blood	Invasive	5-9	Child	Male	GCA_903931585	SAMEA6143146		FD01846184
4025_16	Western	Enteritidis	D1	11	15	9	2016	Blood	Invasive	0-4	Child	Female	GCA_903931455	SAMEA6142035		FD01843899
O008_01	Eastern	Enteritidis	D1	11	1	10	2001	Blood	Invasive	15-19	Child	Female	GCA_903931495	SAMEA6142015		FD01843876
1020_09	Western	Fischerstrasse	V	4270	10	8	2009	Blood	Invasive	0-4	Child	Male	GCA_903931065	SAMEA6142722		FD01845436
4289_14	Western	Give	E1	516	1	12	2014	Stool	Gastroenteritis	0-4	Child	Male	GCA_903930965	SAMEA6141984		FD01843816
3252_14	Western	Give	E1	516	1	9	2014	Abscess	Others	0-4	Child	Male	GCA_903931005	SAMEA6142037		FD01843903
1142/15	Western	Glostrup	C2-C3	NA	1	4	2015	Abscess	Others	0-4	Child	Female	GCA_913082385	SAMEA6142012		FD01843873
2460_13	Western	Grumpensis	G	2060	1	8	2013	Blood	Invasive	0-4	Child	Male	GCA_903931015	SAMEA6142025		FD01843887
0664_15	Western	Gueuletapee	D1	284	1	7	2015	Stool	Gastroenteritis		Adult	Female	GCA_903931155	SAMEA6143123		FD01846153
0289_15	Western	Hessarek	B	4260	1	2	2015	Stool	Gastroenteritis	0-4	Child	Male	GCA_913082355	SAMEA6142039		FD01843905

1076_01	Eastern	Hull	I	1996	1	6	2001	Blood	Invasive	5-9	Child	Male	GCA_903931295	SAMEA6141999		FD01843852
0317_13	Western	Hull	I	1996	1	5	2013	Stool	Gastroenteritis	75-79	Adult	Male	GCA_903931255	SAMEA6141990		FD01843839
2214_18	Western	Hull	I	1996	1	5	2018	Blood	Invasive	10-14	Child	Male	GCA_903931285		ERR7987347	
4586_10	Western	Hull	I	1996	1	5	2010	Blood	Invasive	0-4	Child	Male	GCA_903931375	SAMEA6142023		FD01843885
6650_01	Eastern	I 1,4,12,27:g,m:1,2	B	NA	1	10	2001	Blood	Invasive	10-14	Child	Female	GCA_903931595	SAMEA6143141		FD01846176
1521_08	Western	I 1,6,14,25:y:1,5	H	6046	1	10	2008	Blood	Invasive	0-4	Child	Female	GCA_903930925		ERR7987379	
3653_13	Western	I 6,7:z4,z23:-	C1	4255	1	11	2013	Stool	Gastroenteritis	40-44	Adult	Male	GCA_903931105	SAMEA6141985		FD01843823
1004_07	Western	Landala	S	4262	1	8	2007	Blood	Invasive	30-34	Adult	Male	GCA_903930895	SAMEA6142654		FD01845355
0796_06	Western	Lomita	C1	3039	1	7	2006	Blood	Invasive	45-49	Adult	Female	GCA_903931365	SAMEA6142687		FD01845394
4178_15	Western	Marseille	F	2536	1	10	2015	Blood	Invasive	0-4	Child	Female	GCA_903930985	SAMEA6142034		FD01843898
1585_17	Western	Mbandaka	C1	6049	1	5	2017	Stool	Gastroenteritis	30-34	Adult	Male	GCA_903931125		ERR7987349	
3085_14	Western	Mocamedes	M	1991	1	8	2014	Stool	Gastroenteritis	55-59	Adult	Male	GCA_903930975	SAMEA6141988		FD01843832
1820_14	Western	Neunkirchen	P	4259	1	6	2014	Stool	Gastroenteritis	0-4	Child	Male	GCA_903930915	SAMEA6142007		FD01843864
3220_14	Western	Neunkirchen	P	4259	1	9	2014	Blood	Invasive	0-4	Child	Female	GCA_903930855	SAMEA6142038		FD01843904
1942_14	Western	Neunkirchen	P	4259	1	10	2014	Abscess	Others	5-9	Child	Male	GCA_903930835		ERR7987351	
3541_16	Western	Okerara	E4	651	1	8	2016	Stool	Gastroenteritis	50-54	Adult	Female	GCA_903931145	SAMEA6142022		FD01843883
4278_06	Western	Poona	G	4268	1	10	2006	Abscess	Others	5-9	Child	Male	GCA_903931095	SAMEA6142695		FD01845402
2742_14	Western	Poona	G	308	1	8	2014	Stool	Gastroenteritis	65-69	Adult	Female	GCA_903931135	SAMEA6141996		FD01843848
4159_15	Western	Poona	G	308	1	10	2015	Stool	Gastroenteritis	0-4	Child	Male	GCA_903931075	SAMEA6142021		FD01843882
9710_11	Western	Rubislaw	C1	2642	1	5	2011	Stool	Gastroenteritis	35-39	Adult	Male	GCA_903930875	SAMEA6142000		FD01843854
0525_13	Western	Rubislaw	C1	562	1	9	2013	Stool	Gastroenteritis	0-4	Child	Male	GCA_903931185	SAMEA6141987		FD01843831
0559_17	Western	Seattle	M	NA	1	3	2017	Blood	Invasive	5-9	Child	Female	GCA_903930885		ERR7987352	
1427_09	Western	Stanleyville	B	339	1	10	2009	Blood	Invasive	0-4	Child	Female	GCA_903931275	SAMEA6143134		FD01846169
1058_09	Western	Stanleyville	B	339	1	8	2009	Blood	Invasive	0-4	Child	Female	GCA_903931345	SAMEA6142714		FD01845428
4585_16	Western	Stanleyville	B	339	1	10	2017	Blood	Invasive	0-4	Neonate		GCA_903931315		ERR7987354	
2958_13	Western	Teltow	M	4254	1	10	2013	Blood	Invasive	0-4	Child	Female	GCA_903930865	SAMEA6142010		FD01843871
0123_01	Eastern	Typhimurium	B	19	1	12	2001	Blood	Invasive	10-14	Child	Male	GCA_903931875	SAMEA6143115		FD01846137
0189_01	Eastern	Typhimurium	B	19	1	1	2001	Blood	Invasive	5-9	Child		GCA_903931815	SAMEA6143111		FD01846120
0253_01	Eastern	Typhimurium	B	19	1	1	2001	CSF	Invasive	5-9	Child	Male	GCA_903931695	SAMEA6143127		FD01846160
0781_01	Eastern	Typhimurium	B	19	1	2	2001	Blood	Invasive	0-4	Child	Female	GCA_903931765	SAMEA6143157		FD01846200
1480_02	Eastern	Typhimurium	B	19	1	10	2001	Blood	Invasive	0-4	Child	Female	GCA_903931825	SAMEA6142036		FD01843900
3978_17	Western	Typhimurium	B	19	1	10	2017	Blood	Invasive	45-49	Adult	Female	GCA_903931965		ERR7987356	
1342_06	Western	Typhimurium	B	19	1	11	2006	Blood	Invasive	30-34	Adult	Male	GCA_903931685	SAMEA6142667		FD01845370

0176_14	Western	Typhimurium	B	19	1	1	2014	Blood	Invasive	35-39	Adult	Female	GCA_903931745	SAMEA6142001		FD01843857
1177_07	Western	Typhimurium	B	19	1	9	2007	Blood	Invasive	0-4	Child	Female	GCA_903931785	SAMEA6142720		FD01845434
8696_10	Western	Typhimurium	B	19	1	9	2010	Blood	Invasive	0-4	Child	Male	GCA_903931845	SAMEA6142030		FD01843893
9994_11	Western	Typhimurium	B	19	1	12	2011	Abscess	Others	10-14	Child	Female	GCA_903930795	SAMEA6141994		FD01843846
2408_13	Western	Typhimurium	B	19	1	8	2013	Blood	Invasive	50-54	Adult	Female	GCA_903931675	SAMEA6142018		FD01843879
0165_14	Western	Typhimurium	B	19	1	1	2014	Blood	Invasive	10-14	Child	Female	GCA_903931725	SAMEA6141997		FD01843849
1921_14	Western	Typhimurium	B	19	1	6	2014	Blood	Invasive	0-4	Child	Female	GCA_903931795	SAMEA6141992		FD01843841
4519_14	Western	Typhimurium	B	19	1	12	2014	Blood	Invasive	0-4	Child	Female	GCA_903931805	SAMEA6142026		FD01843888
4516_14	Western	Typhimurium	B	19	1	12	2014	Blood	Invasive	0-4	Child	Male	GCA_903931665	SAMEA6142033		FD01843896
0851_15	Western	Typhimurium	B	19	1	3	2015	Blood	Invasive	45-49	Adult	Male	GCA_903931555	SAMEA6142027		FD01843889
3970_15	Western	Typhimurium	B	19	1	10	2015	Blood	Invasive	50-54	Adult	Female	GCA_903931865	SAMEA6142002		FD01843858
3009_16	Western	Typhimurium	B	19	1	7	2016	Blood	Invasive	0-4	Child	Male	GCA_903931855	SAMEA6142014		FD01843875
4948_17	Western	Typhimurium	B	19	1	11	2017	Blood	Invasive	0-4	Child	Female	GCA_903931755		ERR7987362	
5423_17	Western	Typhimurium	B	19	1	12	2017	Blood	Invasive	0-4	Child	Male	GCA_903931635		ERR7987364	
0171_18	Western	Typhimurium	B	19	1	1	2018	Blood	Invasive	10-14	Child	Female	GCA_903931995		ERR7987338	
3276_18	Western	Typhimurium	B	19	1	8	2018	Blood	Invasive	0-4	Child	Male	GCA_903931925		ERR7987366	
2385_01	Eastern	Typhimurium	B	19	1	7	2001	Blood	Invasive	10-14	Child	Female	GCA_903931895	SAMEA6143156		FD01846199
8488_01	Eastern	Typhimurium	B	19	1	12	2001	CSF	Invasive	0-4	Child	Male	GCA_903931885	SAMEA6143113		FD01846128
0220_17	Western	Typhimurium	B	19	1	2	2017	Blood	Invasive	.	.	.	GCA_903931625		ERR7987367	
5797_16	Western	Typhimurium	B	19	1	12	2016	Blood	Invasive	.	.	.	GCA_913082375		ERR7987369	
4273_17	Western	Typhimurium	B	19	1	10	2017	Stool	Gastroenteritis	5-9	Child	Female	GCA_903931655		ERR7987371	
27696_12	Western	Vinohrady	M	3025	1	12	2012	Stool	Gastroenteritis	15-19	Child	Male	GCA_903931055	SAMEA6142024		FD01843886
4165_15	Western	Vinohrady	M	4258	1	10	2015	Stool	Gastroenteritis	50-54	Adult	Male	GCA_903931215	SAMEA6142028		FD01843890
9063_11	Western	Virchow	C1	841	1	1	2011	Stool	Gastroenteritis	40-44	Adult	Male	GCA_903931245	SAMEA6141986		FD01843830
1967_08	Western	Virchow	C1	181	1	12	2008	Blood	Invasive	0-4	Child	Male	GCA_903931195	SAMEA6142708		FD01845419
8876_10	Western	Virchow	C1	841	1	11	2010	Stool	Gastroenteritis	70-74	Adult	Male	GCA_903931305	SAMEA6142016		FD01843877
0347_07	Western	Virchow	C1	755	1	3	2007	Blood	Invasive	35-39	Adult	Female	GCA_903931165	SAMEA6142688		FD01845395
1156_08	Western	Virchow	C1	841	1	6	2008	Blood	Invasive	0-4	Child	Male	GCA_903931225	SAMEA6142669		FD01845372
5400_08	Western	Virchow	C1	181	1	10	2008	Stool	Gastroenteritis	40-44	Adult	Male	GCA_903931235	SAMEA6142689		FD01845396
1221_09	Western	Virchow	C1	841	1	3	2009	CSF	Invasive	0-4	Child	Male	GCA_903931335		ERR7987372	
2933_14	Western	Wernigerode	D2	2271	1	8	2014	Stool	Gastroenteritis	0-4	Child	Male	GCA_903931085	SAMEA6141991		FD01843840

Supplementary Table 4: Gambian non-typhoidal *Salmonella* serovar distribution and disease prevalence in patients

Serovar	Total	Invasive N (%)	Gastroenteritis N (%)	*Others N (%)	Odds of Invasive vs Gastroenteritis	95% CI	p value
	**93**	**64**	**23**	**6**			
S. Typhimurium	28	26 (30.6)	1 (4.3)	1 (16.7)	15.05	1.91; 118.71	**0.010**
S. Enteritidis	16	13 (20.3)	2 (8.7)	1 (16.7)	2.68	0.52; 7.58	0.220
S. Virchow	7	4 (6.3)	3 (13.0)	0 (0)	0.44	0.13, 2.75	0.314
Other serovars	42	21 (32.8)	17 (73.9)	4 (66.7)	0.17	0.06; 0.50	0.001

*Other = Abscess/pus and urine isolates

Supplementary table 5. Odds of *S.* Enteritidis resistance against other NTS serovars causing disease in The Gambia

Antimicrobials	*S.* Enteritidis n=13	Other serovars n=80	Odds of *S.* Enteritidis vs all serovars	95% CI	P-value
Ampicillin	7	1	59.1	6.51; 536.85	<0.0003
sulfamethoxazole-trimethoprim	6	2	22.5	3.61; 127.05	0.004
Tetracycline	7	2	29.2	5.24; 162.37	<0.0001
Chloramphenicol	2	0	1	NA	NA

Supplementary Table 6: Summary of plasmid and serovar harbouring them in Gambian non-typhoidal *Salmonella* isolates.

Plasmid Type	***occurrence***	***Serovars harbouring plasmids***
IncFIB_S__1	45	Enteritidis, Give, Lomita, Typhimurium
IncFII_S__1	50	Dublin, Enteritidis, Give, Glostrup, Typhimurium
IncFIB_pB171__1_pB171	2	Brandenburg
IncN_1	5	Enteritidis
IncI1_1_Alpha	6	Enteritidis, Poona
IncX1_4	1	Enteritidis
IncX1_1	4	Dublin, Typhimurium, 1,4,12,27:g,m:1,2
ColRNAI_1	2	Poona, Typhimurium
Col_MG828__1	2	Typhimurium, Virchow
pSL483_1	1	Typhimurium
IncI2_1_Delta	2	Poona, Typhimurium
IncL/M_pOXA-48__1_pOXA-48	3	Give, Poona, Typhimurium
repUS21__rep_pWBG764	1	Chester
IncFIB_pKPHS1__1_pKPHS1	4	Give, Neunkirchen
IncFII_SARC14__1_SARC14	3	Neunkirchen
IncFII_p14__1_p14	3	Neunkirchen
IncFII_pRSB107__1_pRSB107	1	Wernigerode
pENTAS02_1	1	Teltow

Supplementary Figure 1. Phylogenetic tree reconstructed from the core-genome analysis of *S.* Typhimurium strains showing plasmids present.

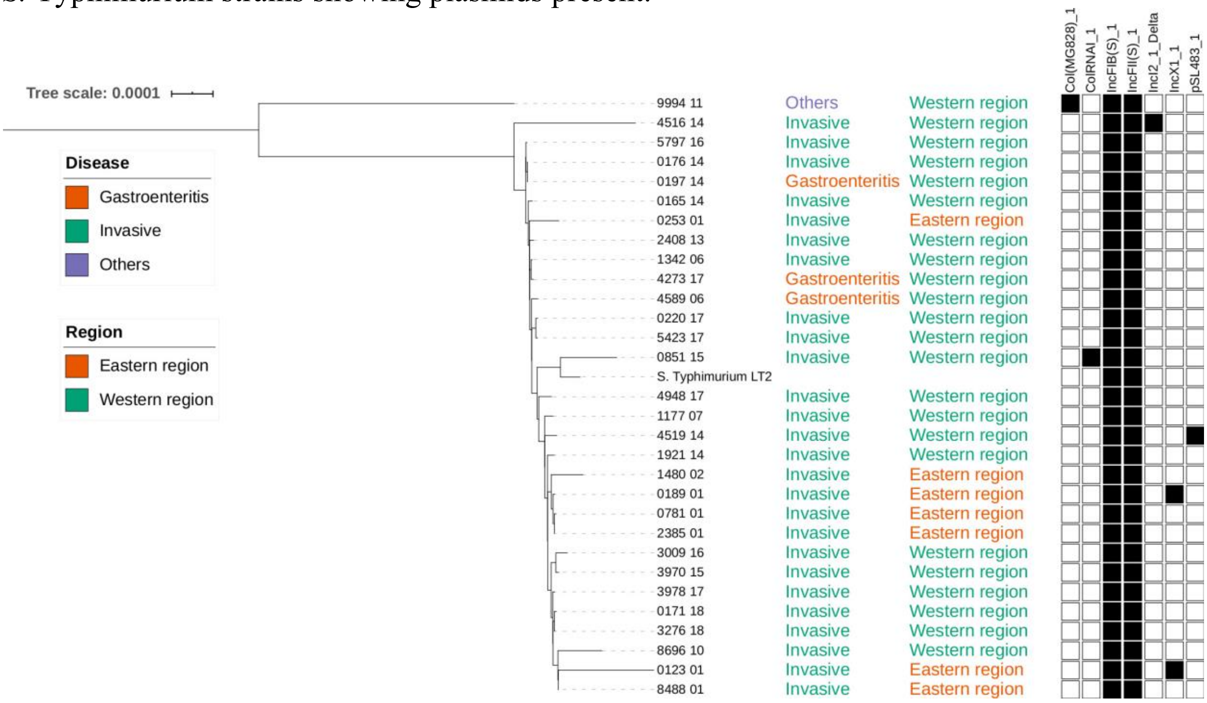

Supplementary Figure 2. Phylogenetic tree reconstructed from the core-genome analysis of *S.* Enteritidis strains showing plasmids present.

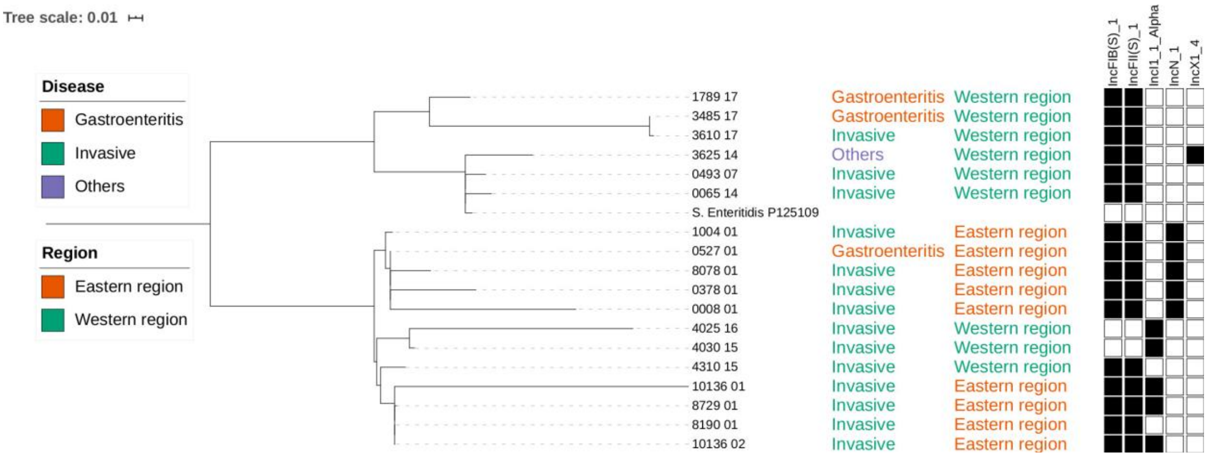

Supplement: Supplementary material 1 [file mgen-8-0785-s001.pdf]
